# Unlocking and accelerating transformations to the SDGs: a review of existing knowledge

**DOI:** 10.1007/s11625-023-01342-z

**Published:** 2023-06-02

**Authors:** Cameron Allen, Shirin Malekpour

**Affiliations:** 1 Monash Sustainable Development Institute, Monash University, Melbourne, VIC Australia; 2 Sustainability Assessment Program, School of Civil and Environmental Engineering, UNSW Sydney, Sydney, NSW 2052 Australia

**Keywords:** Sustainable Development Goals (SDGs), Transformations, Transitions, Leverage points, Tipping points, Acceleration

## Abstract

As we cross the 2030 deadline to achieve the Sustainable Development Goals (SDGs), there is a growing sense of urgency around the need to accelerate the necessary transformations. These encompass a broad range of systems and require fundamental changes in system goals and design. In this paper, we undertake a narrative review of the literature relating to the acceleration of transformations and offer a framework for unlocking and accelerating transformations to the SDGs. While there is no blueprint for acceleration, there is an expanding knowledge base on important dynamics, impediments and enabling conditions across diverse literatures which can help to inform strategic interventions by actors. The emerging literature on positive tipping points and deep leverage points identifies opportunities to rewire systems design so that important system feedbacks create the conditions for acceleration. Transformation takes time and actors will need to build momentum to reorient systems around new goals, informed by knowledge of common policy, technology and behavioural feedbacks that govern system dynamics. Where resistance is strong, actors can seek to augment system design in ways that weaken balancing feedbacks that stabilise existing system configurations and strengthen reinforcing feedbacks that promote emerging system configurations oriented towards the SDGs. Well-designed and sequenced interventions can promote innovation and behaviour change and build and maintain political support. This can build critical enabling conditions and push systems towards large-scale tipping points, paving the way for decisive policy action that is crucial for triggering acceleration. We conclude by highlighting gaps and priorities for further research.

## Introduction

Humanity is currently on an unsustainable trajectory that is unlikely to meet many Sustainable Development Goals (SDGs) by 2030 or even 2050 without decisive action (Rockström et al. 2021; Fanning et al. 2021; Sachs et al. 2021; van Vuuren et al. 2022; Sörgel et al. 2021). Early progress made towards the SDGs has been all but derailed by the COVID-19 pandemic (Nature 2021; Sachs et al. 2021). While devastating, the pandemic has the potential to be a catalyst for a shift towards a sustainable future (Forum for the Future 2020; WBCSD 2020; Linnér and Wibeck 2021; Fioramonti et al. 2022). Such a shift will require transformative change (Chan et al. 2020; Butchart et al. 2019; IPBES 2019; IPCC 2018, 2022a, 2022b; IGS 2019; Sachs et al. 2019), and as we cross the mid-point of the 2030 Agenda, there is growing acknowledgement of the need to accelerate the necessary transformations (UNGA 2019; Markard et al. 2020; Hepburn et al. 2020; Roberts and Geels 2019).

While different fields of research have studied sustainability transformations for some decades, to date, there has been less attention to the deliberate acceleration of transformations to achieve the SDGs (Markard et al. 2020; Linnér and Wibeck 2021; Köhler et al. 2019; Roberts and Geels 2019). The recent elaboration of six key transformations to achieve the SDGs (Sachs et al. 2019; IGS 2019) provides an integrated and coherent organising framework that helps to simplify the 17 goals and focus on their interconnections (Nature 2020). The six transformations are: (1) education, gender and inequality; (2) health, well-being and demography; (3) energy decarbonisation and sustainable industry; (4) sustainable food, land, water and oceans; (5) sustainable cities and communities; (6) digital revolution (Sachs et al. 2019). However, the framework may also downplay the dynamics of transformations, including the inherently political aspects and underlying impediments that continue to prevent progress from accelerating (Brand et al. 2021).

Research on social–ecological systems (SES) transformations (Herrfahrdt-Pähle et al. 2020; Moore et al. 2014; Folke et al. 2021) and sustainability transitions (ST) (Köhler et al. 2019; Loorbach et al. 2017; Markard et al. 2020) perceives acceleration as a phase in the transformation process, shifting from more incremental emergence to rapid diffusion of new ideas, practices and innovations (Markard et al. 2020) which needs to be effectively navigated (Moore et al. 2014; Olsson et al. 2014). In this literature, the concepts of transformation and transition are closely related, where transformation is seen as a fundamental change in system goals, values and paradigm, while transition is the process of systems change from one state to another in a given period of time (IPCC 2022b). In this context, the SDGs transformations involve transitions in a broad range of systems, including for the provision of healthcare, education, food, energy, mobility, housing, water, communications and finance, among others.

Much of the research on governing transitions has focussed on the early stages of experimentation and emergence of innovations in niches (Köhler et al. 2019; Raven et al. 2016; Smith and Raven 2012; Schot and Geels 2008); however, there is an emerging literature on the challenges and mechanisms for the deliberate acceleration of transitions (Markard et al. 2020; Köhler et al. 2019; Roberts and Geels 2019; Roberts et al. 2018). This literature argues that decisive government action through public policy and strategic interventions will be critical for acceleration (Markard et al. 2020; Rosenbloom et al. 2020; Kern and Rogge 2018). Political struggles, conflict and resistance are also likely to be particularly acute in this phase (Markard et al. 2020).

As a global agenda, country context is important. Different countries are at varying stages of transition and have different starting points and priorities. While there is no single blueprint for accelerating transformations (Linnér and Wibeck 2021), the extensive theoretical and empirical literature provides a rich resource to extract key insights on important barriers and enabling conditions, however, with a larger evidence base for Global North contexts (Köhler et al. 2019). Greater attention to system dynamics can increase the policy impact of this research (Alkemade and de Coninck 2021), including through knowledge on how dynamics accelerate transitions and make them reinforcing. Recent contributions highlight strategic interventions where comparatively small efforts can yield large-scale changes towards shared goals (Lenton et al. 2021; Dorninger et al. 2020).

Two systems concepts with practical appeal have gained prominence in the literature, including in recent science-policy assessments (IPCC 2022a, 2022b; IPBES 2019). First, the nascent field of research on social or positive tipping points (Sharpe and Lenton 2021; Stadelmann-Steffen et al. 2021; Lenton et al. 2021; Otto et al. 2020a) highlights opportunities for acceleration through the activation of positive reinforcing feedbacks leading to large-scale systemic shifts towards sustainability (Folke et al. 2021; Sharpe and Lenton 2021). Second, research on system leverage points has seen a resurgence in recent years with new insights for SDGs transformations (Leventon et al. 2021; Linnér and Wibeck 2021; Davelaar 2021; Dorninger et al. 2020; Fischer and Riechers 2019; Abson et al. 2017; O’Brien 2018). This literature underscores that efforts to accelerate transformations may stall without attention to deeper points of leverage associated with system goals, beliefs and paradigms (Leventon et al. 2021; Dorninger et al. 2020; Abson et al. 2017).

However, it is also argued that these systems approaches lack a social science perspective (Alkemade and de Coninck 2021) and should, therefore, be integrated with research on how social change happens (Smith et al. 2020), and how policy processes and feedbacks influence the rate and direction of transitions (Edmondson et al. 2019; Kern and Rogge 2018; Kern et al. 2019). Insights from these diverse literatures can help to shed light on key impediments and enabling conditions for accelerating transformations to the SDGs.

In this paper, we review and synthesise research on the acceleration of transformations with the aim of providing insights for implementation of the SDGs. We first review important impediments and enabling conditions for acceleration from the sustainability transformations literature and then integrate insights on acceleration from recent literature on positive tipping points, the leverage points perspective and on political dynamics of major policy reforms. We then synthesise key insights from these different literatures in the context of accelerating transformations to achieve the SDGs and highlight important research gaps. The main contribution of the paper is theoretical and conceptual—integrating diverse and dispersed insights to advance knowledge on acceleration.

Relevant literature was initially identified through a keyword query^1^ of the Web of Science database (*n* = 529) with keywords corresponding to the SDGs and sustainable development, transition and transformation, as well as key concepts associated with acceleration, tipping points, leverage points, system dynamics, feedbacks and policy processes/mixes. Given that the literature on sustainability transformations is extensive, we prioritised papers that specifically addressed SDGs transformations and included key concepts of interest. All articles were screened for relevance by reviewing their title, keywords and abstract to identify papers that provided a review or synthesis of the literature on transformations to sustainable development, or important theoretical, empirical, or conceptual advancements (*n* = 51). From this starting point, we undertook a more detailed review of shortlisted papers and snowballed additional highly cited and highly relevant articles in the predominantly academic literature.

## Accelerating transformations to sustainable development

### Research on sustainability transformations

Many different research fields are advancing concepts and empirical analysis on transformations to sustainable development and there have been several reviews in recent years (Feola 2015; Scoones et al. 2020; Hölscher et al. 2018; European Environment Agency 2018; Linnér and Wibeck 2019; Salomaa and Juhola 2020). This research suggests that deliberate transformations to sustainable development can take various systemic, structural and emergent forms (Scoones et al. 2020) with many potential pathways (Stirling 2015), involving different actors and degrees of agency in terms of enabling and navigating change (European Environment Agency 2018).

Of particular interest for this review is the perspective that it is possible to influence the speed and direction of transitions towards sustainability (Köhler et al. 2019) and that strategic interventions and public policy can play a key role in this regard (Edmondson et al. 2019; Geels 2019; Markard et al. 2020). Aligned with this perspective, systemic approaches to transformation include research fields associated with ST and SES transformations and advocate for the deliberate acceleration of innovations and progressive policy for managing social, technological and ecological transitions (Scoones et al. 2020). Both strands of the literature describe sustainability transformations as multi-level, multiphase and cross-scale processes; however, they have different points of departure (Olsson et al. 2014).

In SES theory, sustainability transformations are understood as shifts that fundamentally alter human and environmental interactions and feedbacks with attention to thresholds and non-linear dynamics (Olsson et al. 2006, 2014; Holling and Gunderson 2002; Walker et al. 2004). In contrast, in the ST literature, sustainability transitions require radical shifts to new kinds of socio-technical systems in response to persistent societal challenges by rapidly scaling up new technologies, institutions and routines (Loorbach et al. 2017, Geels 2002, 2011, 2019; Köhler et al. 2019). The different strands of research have become more integrated over time by combining theoretical and empirical insights (Loorbach et al. 2017; Olsson et al. 2014; Herrfahrdt-Pähle et al. 2020; Folke et al. 2021; Geels 2019). Important commonalities include notions such as path dependencies, experiments, tipping points, and multiple levels, pathways and phases (Loorbach et al. 2017). Typical phases from the SES literature involve preparing the system for change, navigating change and institutionalising or building resilience of the new trajectory (Moore et al. 2014; Feola 2015). In the ST literature, key phases include predevelopment/emergence, acceleration and stabilisation (Markard et al. 2020; Kivimaa et al. 2019). Dominant system configurations or regimes can become stressed or destabilised as broader societal contexts change (landscape pressures) and new radical alternatives develop and emerge (niche pressures), which can lead to systemic reconfiguration (Loorbach et al. 2017). However, path dependencies often supress these transition processes, suggesting an important role for governance actors (Hölscher et al. 2019).

**Fig. 1:**
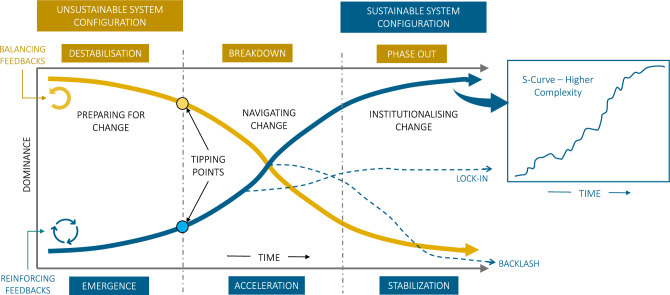
An ideal-type sustainable development transition. Rising and declining S-curves across three phases of transformation. Inertia and non-linear dynamics are shaped by balancing (negative) and reinforcing (positive) feedbacks. Tipping points demarcate the inflection point between emergence and acceleration. Dotted lines depict alternative pathways (lock-in, backlash). In the right panel, an ideal transition pathway is rarely smooth and faces many impediments

Successful transformations from unsustainable to sustainable system configurations (Fig. 1) often entail breaking down resilient structures and processes while also building new desirable ones (Herrfahrdt-Pähle et al. 2020; Elmqvist et al. 2019; Markard et al. 2020; Anderson et al. 2019). Figure 1 presents such a stylised transition, whereby the dominant system configuration (*y*-axis) shifts from unsustainable to sustainable over time (*x*-axis).

An ideal-type or successful transition pathway for a sustainable system configuration including sustainable technologies, institutions and practices can be simply visualised through an S-shaped curve (Fig. 1—blue curve) corresponding to the phases of emergence, acceleration and stabilisation. The S-curve is common in empirical research on technology and innovation diffusion (Markard et al. 2020; Grubb et al. 2021; Rogers 2003; Loorbach et al. 2017) and is also a familiar pattern in systems research where it is often used to represent the life of a system (Davelaar 2021) or a social–ecological transformation (Herrfahrdt-Pähle et al. 2020; Folke et al. 2021; Elmqvist et al. 2019).

The rise of a new system configuration is also mirrored by the decline of existing unsustainable technologies, institutions and practices, for example, where the rise of renewable energy or regenerative agricultural system configurations mirror the decline of fossil fuel energy or industrial agriculture systems configurations. This can be depicted through a declining S-curve with corresponding phases of destabilisation, breakdown and phase-out (Fig. 1—yellow curve). The use of two S-curves—one declining and one rising—takes the form of an ‘X-curve’ which represents the dual process of growth and decline in transformation (Sharpe et al. 2016; Davelaar 2021; Herrfahrdt-Pähle et al. 2020; Markard et al. 2020; Hebinck et al. 2022).

The shape of the S-curve simplifies complex system dynamics with longer periods of stability punctuated by rapid non-linear change. From a systems perspective, balancing and reinforcing feedback loops play an important role in these dynamics (Stroh 2015; Sterman 2012; Sharpe and Lenton 2021). Dominant balancing feedbacks stabilise existing system configurations and make them resistant to change (Fig. 1—yellow curve). In contrast, reinforcing feedbacks (Fig. 1—blue curve) can drive rapid non-linear system shifts once important tipping points are crossed. In reality, transitions may oscillate as a result of different feedback effects and perturbations (e.g. crises) which can destabilise systems and result in complex pathways that are rarely smooth (Fig. 1—inset box).

A successful transition pathway is far from guaranteed (Geels and Schot 2007; de Haan and Rotmans 2011). For example, promising emerging innovations can be met with a range of impediments (see “Critical barriers and enabling conditions for accelerating transformations”), resulting in pathways that fail to fundamentally alter the status quo, such as ‘lock-in’ or ‘backlash’, among others (Fig. 1, dotted blue lines). For example, such pathways can be seen in countries where carbon prices or mandatory greenhouse gas reduction targets have been legislated to accelerate energy system transitions but then subsequently repealed due to strong lobbying from powerful interests (Rabe 2018). Knowledge of common system feedbacks and impediments can help to promote successful efforts to deliberately steer systems towards fundamentally new configurations.

### Critical barriers and enabling conditions for accelerating transformations

The literature identifies a broad range of impediments and barriers to transformation as well as important enabling conditions, and notes that these will vary across the different phases of transformation (Kivimaa et al. 2019; Safarzyńska et al. 2012; Grin et al. 2010; Köhler et al. 2019; Olsson et al. 2014). Of particular interest for this review are important barriers and enabling conditions associated with the acceleration phase of transformations, as well as the emergence phase which needs to be transgressed to reach acceleration (Table 1).

**Table 1:** Summary of common barriers and enabling conditions during emergence and acceleration phases of transformations as identified in the literature

Barriers/challenges	Enabling conditions	Sources
Emergence		
Lack of agreement on the need for change Disinformation Narrow/traditional problem framings Social norms against change Fear of change Lack of diversity, human capital and ideas Deficits in agency, representation and power imbalances Absence of leadership, trust and good social relations Weak institutions; political and economic instability	New problem definitions New framings (e.g. as systems challenge) New narratives Awareness of the need for change Protected spaces for innovation, experimentation and coproduction Opportunities for contestation Leaders who can build trust, form coalitions and communicate compelling narratives Innovation networks, niche lobbies Independent media Policies promoting innovation -e.g. R&D, targets, missions, incentives, pilots Crises or anticipated risks	Herrfahrdt-Pähle et al. (2020), Köhler et al. (2019), Mossberg et al. (2018), Schot and Steinmueller (2018), Loorbach et al. (2017), Folke et al. (2010), Olsson et al. (2006), Smith et al. (2020), Avelino et al. (2016), Petridou and Mintrom (2021), Mintrom (2019), Cosens et al. (2014), Olsson et al. (2010), Patterson et al. (2017), Markard et al. (2021), Markard (2018), Raven (2007), Musiolik et al. (2012)
Acceleration		
Techno-economic lock-ins from sunk investments, infrastructure, technology maturity Social and cognitive lock-ins from dominant routines, mindsets and practices Institutional and political lock-ins from existing regulations, standards and policy networks Resistance from incumbents and vested interests Shifting from single innovations to whole systems configuration Access to scalable finance—risk–reward profiles, market design, high finance costs Hands-off or market failure policy paradigm—policy vacuum	Changing policy paradigms/goals—i.e. to a proactive state shaping markets, launching larger missions, investing in infrastructure, redirecting finance Maturation of niche innovations Mobilisation of coalitions and social movements Shifts in public opinion and pervasive narratives; positive niche discourses Dissatisfaction—negative regime discourses Policy entrepreneurs; support from powerful actors Vertical and horizontal coordination New business models External shocks or crises; political regime destabilisation Weakening regime lobbies; united niche lobbies Changing stakeholder access to institutions, policy networks	Sharpe and Lenton (2021), Geddes and Schmidt (2020), Markard et al. (2020), Geels (2018), Rosenbloom et al. (2020), Markard (2018), Markard et al. (2021), Meadowcroft (2009), Normann (2017), Geels (2019), Klitkou et al. (2015), Di Gregorio et al. (2019), Markard et al. (2016), Hess (2014), Meijerink (2005), Meckling et al. (2015), Raven et al. (2016), Roberts and Geels (2018), Rosenbloom et al. (2016), Roberts and Geels (2019), Capoccia and Kelemen (2007), Avelino et al. (2016), Gunderson et al. (2017), Herrfahrdt-Pähle et al. (2020), Schmidt and Sewerin (2017), Petridou and Mintrom (2021), Mintrom (2019)

The emergence (destabilisation) phase (Fig. 1) is described as a dynamic equilibrium where change is incremental and the status quo does not visibly change (Rotmans et al. 2001) but experimentation takes place (Safarzyńska et al. 2012) arising in a combination and conflict between niche ideas and a reluctance to change existing system (or regime) configurations (Kivimaa et al. 2019). For example, problems with congestion, air pollution and deteriorating environments have prompted cities to experiment with nature-based solutions (Bai et al. 2016). Social and governance innovations are also emerging in response to key societal challenges, for example, universal basic income to address inequality, cooperative housing organisations to provide affordable social housing and member-owned credit cooperatives to support local farmers (Pel et al. 2020).

Important enabling conditions (Table 1) in the emergence phase include new problem framings and an awareness of the need for change, protected spaces for experimentation with innovations, policies promoting innovation and alliances that organise actors towards a common vision and goals (Herrfahrdt-Pähle et al. 2020; Köhler et al. 2019; Mossberg et al. 2018; Schot and Steinmueller 2018; Loorbach et al. 2017). Crises and new scientific knowledge of problems (e.g. climate change) can trigger experimentation with new practices which help to prepare the system for change (Folke et al. 2021; Herrfahrdt-Pähle et al. 2020).

As much of the literature on barriers and enablers corresponds to countries in the Global North, further expanding empirical studies to countries in the Global South is needed (Köhler et al. 2019). Existing studies find that the main frameworks, concepts and dynamics remain relevant in different country contexts; however, some countries may face additional impediments including weak institutions and innovation systems, conflict and instability, market imperfections, clientelism and corruption, and inequalities in accessing finance and global markets (Ramos-Mejía et al. 2018; Hansen et al. 2018; Wieczorek 2018). Technological and social innovations will also be important to address development priorities such as poverty alleviation (Ramos-Mejía et al. 2018; Romijn and Caniëls 2011), as will building institutional capabilities (Cid et al. 2020).

In the acceleration (breakdown) phase **(**Fig. 1**)**, the dominant configuration changes as a result of self-examination or in response to bottom–up or top–down pressures (Grin et al. 2010). As new ways of thinking, doing and organising emerge in different systems, they may reach a tipping point where they are adopted or intensely advocated and ‘take-off’ (Smith and Raven 2012; Geels 2005; Gorissen et al. 2018). Both slow moving trends (e.g. demographics, ideologies and accumulation of greenhouse gas emissions) and sudden shocks (e.g. economic crises, pandemics and extreme events) can also start to weaken or disturb an existing system (and its stabilising feedbacks) and create windows-of-opportunity for new practices, governance systems or value orientations to accelerate and rapidly become dominant (Olsson et al. 2014, 2006; Folke et al. 2021).

However, due to a range of impediments, there is no guarantee that emerging technologies, behaviours or practices will accelerate (Table 1). Several common ‘lock-in’ mechanisms mean that progress is often incremental and may take many years to accelerate, if ever (Klitkou et al. 2015; Geels 2019). These lock-ins may prevent emerging sustainable innovations from significantly altering dominant system configurations, resulting in suboptimal pathways (Fig. 1, dotted blue lines).

First, techno-economic lock-ins result from sunk investments (e.g. in competencies, factories and infrastructures) that create vested interests against change, as well as the low cost and high-performance characteristics of existing technologies which benefit from economies of scale and decades of learning-by-doing improvements. For example, the cost-competitiveness of renewable energy technologies compared with conventional energy technologies has a strong influence on their adoption and the pace of the transition. Scaling up emerging innovations requires large-scale investment which may be hindered by poor risk–reward profiles or by high financing costs (particularly for developing markets), or inertia in market design and business models (Sharpe and Lenton 2021; Geddes and Schmidt 2020). During acceleration, there is often a marked shift from promoting single innovations in specific sectors to whole systems change, requiring complementary technologies, infrastructure or capabilities which may be lacking (Markard et al. 2021; Victor et al. 2019). For example, supporting infrastructure such as electricity grids have been designed in many cases to support large, centralised power stations which may impede the expansion of decentralised renewables. Similarly, adequate charging infrastructure may be needed before a transition to electromobility can accelerate.

Second, social–behavioural lock-ins result from dominant routines, shared mindsets, user practices and lifestyles which become organised around particular technologies, practices and behaviours. For example, people may resist shifting from meat-based to plant-based diets, using sustainable public transport rather than personal vehicles, or reducing the consumption of ‘junk food’ or ‘fast fashion’. Such changes are culturally and politically difficult because consumption is closely related to issues of identity, freedom and established practices around work and family (Markard et al. 2020) and highly affluent consumers often drive consumption norms (Wiedmann et al. 2020).

Third, institutional–political lock-ins result from existing regulations, standards, policy networks or institutions that favour incumbents and create an uneven playing field, whereby vested interests use their access to policy processes to resist or water down policy change and innovation (Normann 2017; Geels 2019; Klitkou et al. 2015). For example, incumbent firms may use powerful and well-connected industry lobbies to influence regulations, market rules and government subsidies in their favour which can entrench unsustainable business models, as has been seen in electricity markets that favour centralised fossil fuel generators and make market entry harder for decentralised renewable sources (Hudson 2020).

As systems transitions accelerate, they can broaden in scope, including through impacts on other systems or policy objectives, as well as geographically such as through spill over effects on other regions and countries (Markard et al. 2020). These may result in conflicts or trade-offs between competing objectives which can exacerbate political conflicts. Policy mixes need to manage wider systems configurations and deal with cross-sectoral interlinkages and integration (e.g. between power generation, transport and heating) (Markard et al. 2020; Geels 2018; Rosenbloom et al. 2020; Markard 2018). Ultimately, the time, capacity and resources needed to accelerate whole systems change can be prohibitive (Geels 2018; Markard et al. 2020).

A common theme in research on acceleration is the need for a stronger and more proactive role by governments in shaping markets, stimulating innovation, investing in infrastructure, setting targets, launching larger missions and regulating businesses (Markard et al. 2020, Roberts and Geels 2019; Geels 2019; Sachs et al. 2019; Mazzucato 2018; Kattel et al. 2018). However, policymakers are often resistant to policy change as they are influenced or even captured by vested interests, tied up by lobby groups and other power structures, or lack the capacity, resources and incentives to act (Otto et al. 2020b; Markard et al. 2020). Such systems resistance can entrench existing unsustainable configurations (Fig. 1—yellow curve) and will likely come from declining industries or incumbent firms and actors, as well as from particular localities or social groups, unions and workers whose jobs are at stake or that will suffer more from decline and phase-out than others (Markard et al. 2020).

Policymakers are, therefore, unlikely to act unless critical conditions for acceleration are in place (Roberts and Geels 2019). Empirical research suggests that important conditions for stronger policy support are associated with changing external pressures from business, the mass public and technology advancements, as well as changes in policy regimes resulting from new problem definitions, new institutional arrangements and support from powerful actors (Roberts and Geels 2019) (Table 1).

## System dynamics insights for accelerating transformations

The speed of transformations and how they can be accelerated remains an important and emerging topic in transformations research (Köhler et al. 2019; Markard et al. 2020; Roberts and Geels 2019; Roberts et al. 2018). There have been recent calls to increase the policy relevance and impact of this research through greater emphasis on system feedbacks and how system dynamics can accelerate transitions and make them self-reinforcing (Edmondson et al. 2019; Kern et al. 2019; Alkemade and de Coninck 2021). Of particular interest is research identifying strategic interventions that leverage important system feedbacks that result in rapid, large-scale changes (IPCC 2022a, 2022b; IPBES 2019). Using empirical evidence of acceleration in domains such as renewable energy and electric vehicles, a nascent literature on positive tipping points is exploring the triggers, dynamics and enablers of these rapid shifts and yielding new insights for acceleration (Sharpe and Lenton 2021; Otto et al. 2020a). At the same time, there is an acknowledgement that attention to deep leverage points can also unlock major transformative shifts (Leventon et al. 2021; Dorninger et al. 2020; Abson et al. 2017). However, any deliberate attempt to accelerate transformations will also need to consider and navigate common impediments, political dynamics and policy processes and feedbacks (Edmondson et al. 2019; Kern et al. 2019; Kern and Rogge 2018; Smith et al. 2020).

### Positive tipping points for accelerating transformations

Tipping points demarcate the inflection point between emergence and acceleration, shifting from incremental to rapid exponential progress and moving up the S-curve (Fig. 1) (Loorbach et al. 2017; Markard et al. 2020). In research on STs, tipping points have received some limited attention to date (Köhler et al. 2019), for example reflecting the point when decisive political action accelerated the transition (Roberts and Geels 2019). In the SES literature, Milkoreit et al. (2018) define them as points where a small quantitative change inevitably triggers a non-linear change in a system, driven by self-reinforcing positive feedback mechanisms. While originally used in this literature to describe critical climate or ecological thresholds (Lenton et al. 2008; Scheffer et al. 2001), the concept has recently been extended to research on social tipping dynamics (Otto et al. 2020a; Winkelmann et al. 2022; Stadelmann-Steffen et al. 2021), positive tipping points (Tàbara et al. 2018; Nyborg et al. 2016; Lenton 2020; Lenton et al. 2021; FOLU 2021), socio-economic tipping points (Van Ginkel et al. 2020), sensitive intervention points (Farmer et al. 2019) and upward tipping cascades (Sharpe and Lenton 2021). These concepts are closely related, and different social actors can intentionally identify and trigger ‘positive tipping points’ to accelerate progress and achieve transformative change (Lenton et al. 2021).

A positive tipping point (TP) is the point when a certain belief, behaviour or technology spreads from a minor tendency to a major practice over a short period of time (Otto et al. 2020a; Stadelmann-Steffen et al. 2021). Important TPs identified in the literature include critical mass (e.g. adopting a technology, social norm, idea and innovation) and critical price (e.g. of an existing versus clean technology or practice) (Zeppini et al. 2014). Reinforcing feedbacks that propel tipping effects have been identified as including social contagion (positive experience), increasing returns to adoption (learning by doing, economies of scale, technological reinforcement), information cascades and certain types of ecological feedbacks, among others (FOLU 2021; Lenton et al. 2021).

A critical mass of people can tip most (or all) of the population to adopt a new innovation, practice, norm or behaviour (Rogers 2003). For example, if consumers start shopping for local and organic produce, electric vehicles or energy-saving appliances, then suppliers will increasingly produce or provide them. This can gradually spread through social contagion effects and result in increased economies of scale and competitiveness which may reach a tipping point, beyond which adoption rapidly accelerates and becomes the new norm. Centola et al. (2018) suggest that 20–30% of a population becoming engaged in an activity can be sufficient to tip the whole society, while other documented instances show that a 17–20% market or population share can be sufficient (Koch 2011; Otto et al. 2020a). For complex contagion, ~ 25% of a population is used as a rough rule of thumb for tipping a change in social convention (Centola et al. 2018; Rogers 1962; Lenton et al. 2021). Research on the typical temporal and spatial scales of social tipping suggests that these commonly exist on the national or sub-national level, and transitions often occur on the scale of years to decades (Winkelmann et al. 2022). However, crossing TPs in a small number of countries can also trigger upward-scaling tipping cascades to the regional and global scales (e.g. in the case of renewables and electric vehicles) (Sharpe and Lenton 2021).

An example of this can be seen where pricing policies and targeted investments bring clean technologies below the threshold of cost-parity with fossil fuel technologies. This can trigger reinforcing feedbacks, as seen in the recent rapid growth in many countries of solar and wind installations and a shift away from fossil fuel generators (Sharpe and Lenton 2021). Policies for the mandatory phase-out of fossil fuel generation can further accelerate this transition. Global developments have also been important, where research and development and market support policies in some countries (e.g. feed-in tariffs in European countries) have been important in driving initial global demand, while firms (e.g. in China) with state investment have scaled up production and delivered the significant cost reductions needed for reaching price parity (Quitzow 2015).

An important insight from this literature is that a mix of well-sequenced ‘tipping interventions’ can push a system across TPs and accelerate the shift to a new state (Tàbara et al. 2018). These interventions are diverse, but generally come in the form of new policies, new technologies and innovations, and new behavioural norms (Stadelmann-Steffen et al. 2021; Farmer et al. 2019). For example, the rapid phase-out of chlorofluorocarbons (CFCs) benefited from several important tipping dynamics in response to the initial shock caused by scientific knowledge of their effects on the ozone layer. This included political tipping dynamics caused by public concern and unilateral banning of CFCs in several countries in the 1970s, triggering technological tipping dynamics through the development, standardisation and scale-up of replacement technologies, and finally further behavioural tipping as the public shifted their consumption patterns as they became increasingly aware of the risks to human health (Stadelmann-Steffen et al. 2021).

This highlights that decisive policies and actions by powerful actors can trigger positive tipping dynamics associated with reinforcing feedbacks (Winkelmann et al. 2022; Stadelmann-Steffen et al. 2021). In the context of decarbonisation, important tipping interventions include removing fossil fuel subsidies, incentivising decentralised energy generation and divesting from assets linked to fossil fuels (Otto et al. 2020a). The sequencing of interventions is also critical to ensure that early interventions create enabling conditions for positive reinforcing feedbacks that drive acceleration. These enabling conditions include improving economic competitiveness, performance, social acceptance, accessibility and capability to adopt new innovations (Lenton et al. 2021; FOLU 2021). For example, in the case of shifting societies towards a planetary health diet, early interventions might include investment in research and innovation to improve performance of alternatives (FOLU 2021). This could be followed by public procurement, market incentives and awareness campaigns to generate demand, build social acceptance and improve economic competitiveness and access, and finally major market interventions (e.g. taxes, regulations) to rapidly scale-up adoption (FOLU 2021).

### Deep leverage points (LPs) for accelerating transformations

Leverage points (LPs) are places in complex systems where a small shift may lead to fundamental changes in the system as a whole (Meadows 1999). The concept is more abstract and has stronger coverage in systems theory and literature (O’Brien 2018; Fischer and Riechers 2019; Abson et al. 2017; Chan et al. 2020; Leventon et al. 2021; Linnér and Wibeck 2021; Dorninger et al. 2020; Davelaar 2021; Birney 2021; Angheloiu and Tennant 2020; Koskimäki 2021; Fortnam 2019; Wigboldus and Jochemsen 2021; Kretschmer and Kahl 2021; Kieft et al. 2020). LPs range on a scale from shallow (places where interventions are relatively easy to implement yet bring about little change to the overall system) to deep (places that are more difficult to alter but potentially result in transformative change) (Davelaar 2021; Abson et al. 2017; Koskimäki 2021).

**Fig. 2:**
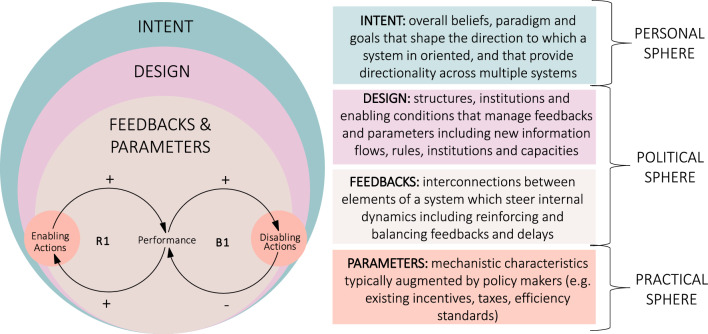
Nested hierarchy of leverage points ranging from deep to shallow and various categorisations (Dorninger et al. 2020; Abson et al. 2017; Meadows 1999; O’Brien 2018). Refer to Meadows (1999) for a description of the original twelve LPs

Much of the recent literature builds on or simplifies the original framework of twelve LPs developed by Meadows (1999), often identifying three or four main categories or ‘spheres’ (Fig. 2) (Abson et al. 2017; Dorninger et al. 2020; Fischer and Riechers 2019; O’Brien 2018). These form a nested hierarchy, whereby the system paradigm and intent (or goals) shape the physical and institutional design of the system, which in turn determines the feedback that the system provides regarding system functioning and finally the type of parameter that can, or should be, adjusted to shift systems towards sustainability (Dorninger et al. 2020; Abson et al. 2017). This nested hierarchy is depicted in Fig. 2, drawing on the existing literature.

As noted in the earlier Fig. 1, reinforcing and balancing feedback loops are known to play an important role in the rapid transition of systems between dynamically stable states (Leventon et al. 2021; Meadows 2008). In the field of systems dynamics, these feedback loops are the two basic structures that describe how systems evolve over time, with more complex non-linear dynamics resulting from combinations of these feedbacks (Sterman 2012).

First, reinforcing feedback loops (Fig. 2—R1 loop) drive the processes of (exponential) growth in systems and explain why some systems have the tendency to amplify interventions where even a small change to a parameter within such loops can have drastic results. Returning to Fig. 1 (blue curve) and the literature on positive tipping points, such reinforcing feedbacks are important for unlocking acceleration dynamics in the rise of new technologies, innovations and behaviours which can stabilise over time as new sustainable system configurations.

Second, balancing feedback loops (Fig. 2—B1 loop) create the dynamics of stability and equilibrium and are often dominant in systems but more difficult to discern and influence (Stroh 2015). These loops give a system the tendency to return to the position it started from, and identifying these loops is important to understand why and how systems delay, dilute or defeat interventions (Sterman 2012). Returning again to Fig. 1 (yellow curve), balancing feedbacks explain inertia and path dependency in existing unsustainable system configurations which are associated with the various system lock-ins and impediments described above.

Understanding system feedbacks is important for those seeking to accelerate transitions; however, the LPs perspective also places this in a broader context of systems design and intent (Fig. 2). Ultimately, the dominant feedbacks in a system are in dynamic equilibrium around the goals that the system is already achieving (Stroh 2015), highlighting why fundamentally changing the system intent is key for transformational change (Chan et al. 2020; Fischer and Riechers 2019; Abson et al. 2017). In Fig. 2, goals form part of the system intent (outer sphere) along with underpinning beliefs and worldviews (or ‘paradigm’) of actors. This shapes the emergent directionality to which a system is oriented (Abson et al. 2017; Meadows 1999). While not all actor goals are necessarily aligned, they collectively point in a certain direction. This orientation is an emergent property of systems and ultimately influences system design (Fig. 2, middle sphere), including the social structures and ‘rules of the game’ as formalised in laws, procedures, standards, incentives, codes of conduct, etc. While there are generally no major physical constraints to changing systems intent, significant changes are often met with substantial political resistance (Kieft et al. 2020). The deeper the leverage point is, the greater the resistance (Meadows 2008).

Based on this logic, both the dominant unsustainable and new sustainable system configurations (Fig. 1) would comprise the various system elements identified in Fig. 2—i.e. they have their own dominant goals or intent, which in turn influence their system design and dominant feedbacks. Policy makers and other actors may seek to enable systems transitions by adjusting different parameters (e.g. policies or incentives) which may augment systems performance, particularly if they target important feedbacks. However, these are unlikely to deliver systems transformation unless they also lead to a fundamental change in system goals and design and the dominance of important feedbacks over time.

Despite their importance for systems change, Dorninger et al. (2020) find that deeper LPs are rarely addressed in empirical studies and there is limited practical guidance on how these shifts can be deliberately accelerated. Emerging research on ‘deep transitions’ suggests that major paradigm shifts have occurred over successive great surges of development lasting around 50–60 years (Perez 2013), involving changes in dominant ‘paradigmatic principles’ which guide the creation and expansion of all systems in a similar direction (Kanger and Schot 2019). The dominance of these principles is not static and shifts occur as new principles emerge from different systems and align across systems, fundamentally changing the overall directionality of all systems (Schot and Kanger 2018). This highlights that fundamental change in paradigms or system intent is possible or even inevitable over longer timeframes.

More knowledge is needed on how to deliberately shift emergent paradigms and goals, the interactions between shallow and deep leverage points, and the potential to sequence or combine interventions and how they influence or trigger one another (Leventon et al. 2021; Abson et al. 2017). Research linking LPs to sustainability transitions suggests that shallower interventions (e.g. parameters) can build support for deeper shifts over time (Kieft et al. 2020; de Gooyert et al. 2016). This places emphasis on the design of interventions, whereby combinations of shallower interventions in a policy mix can play an important supportive role, and indeed are even necessary to reach the potential of a deeper intervention (Kieft et al. 2020). Similarly, nominal changes in goals may have little effect if institutions and infrastructure (systems design) are not adapted to align to these, which may take time to manifest.

### Political dynamics of acceleration—the role of actors and policy feedbacks

Research on past transitions underscores the critical role of decisive government action (e.g. major policy reforms) in triggering rapid systems change and that such action will likely face strong political resistance (Johnstone and Newell 2018; Markard et al. 2020; Roberts and Geels 2019; Geels 2019; Roberts et al. 2018; Kern and Rogge 2016; Edmondson et al. 2019). A key question for actors seeking to accelerate transformations relates to the political conditions, actor roles and processes that lead to the necessary decisive action in different country contexts (Dutt 2022). The TPs and LPs perspectives identify important system traits and strategic interventions; however, they do not explain in which situations it is feasible to overcome resistance to these interventions (de Gooyert et al. 2016).

Policy process theories highlight that major policy change involves competing coalitions supporting and resisting change, which are held together by shared beliefs, goals or narratives/discourses (Sabatier 1988; Geels and Penna 2015; Markard et al. 2016; Rosenbloom et al. 2016; Hajer 1995; Marsh and Rhodes 1992). Dominant coalitions tend to be stable over time but can shift as a result of changing values or shared discourses, or through changes to policy network structures and processes of alliance building, negotiation, bargaining and compromise (Marsh and Rhodes 1992; Normann 2017). Struggles between ‘coalitions for change’ and powerful incumbent coalitions involve contests for power played out in significant part through arguments about the ‘best’ narrative or story (Fischer 2003; Rosenbloom et al. 2016). The appeal and salience of narratives varies during transitions based on how they are perceived, how well they resonate and the credibility of the narrators (Roberts 2017; Rosenbloom 2018).

Dominant narratives (or ‘policy paradigms’) play a crucial role in shaping socio-political interpretations of problems, goals and solutions (Rosenbloom et al. 2016; Van Der Leeuw 2020; Hermwille 2016). As with the systems in which they are embedded, policies are generally marked by long periods of stability. Major policy change (e.g. through shift in policy paradigm) is more likely when windows-of-opportunity open through important developments within the politics stream (e.g. a change in government) or by the emergence of significant problems becoming visible through focussing events in the problem stream (e.g. crises) (Kingdon 2003; Normann 2015; Baumgartner et al. 2018). During these moments, actors such as policy entrepreneurs can achieve policy change through alternative framing, public discussions and lobbying (Mintrom 2019). This could fundamentally change system goals and design (Fig. 2), triggering a rapid shift towards a sustainable system reconfiguration (Fig. 1). However, windows can be missed in the absence of well-developed alternative goals, narratives and solutions that can be pushed at opportune moments (Boin et al. 2009).

The creation of new policies also occurs in the context of existing policies, providing resources and incentives for different political actors that result in policy feedback effects which alter the capacities and interests of actors (Pierson 2000; Pierson 1993). These are described in the literature as positive (reinforcing) or negative (balancing) feedbacks, and include socio-political, fiscal and administrative feedbacks (Edmondson et al. 2019). Reinforcing policy feedbacks occur when well-designed policies bolster their own bases of political support and endure over time. Such feedbacks can help drive systems transitions through acceleration to institutionalisation, where they stabilise as new system configurations (Fig. 1). For example, positive policy feedbacks may be generated where new policies include visible benefits for the mass public (e.g. through beneficial social welfare or tax reforms) (Kern and Rogge 2018), produce a large and powerful coalition seeking reform (e.g. including government, business, unions and other powerful actors), or successfully encourage target groups to make large sunk investments (e.g. in pollution control technologies) (Jordan and Matt 2014).

Balancing policy feedbacks are associated with backlash dynamics and occur when a policy instrument creates forces that counteract its effect and return the whole system to something like its original position (Jordan and Matt 2014). They may, for example, deliver suboptimal transition pathways such as ‘lock-in’ or ‘backlash’ as depicted in Fig. 1. Balancing feedbacks can also result from poorly designed and implemented policies which undermine political momentum and limit the transformative potential of policy reforms (Jacobs and Weaver 2015; Kern et al. 2019; Edmondson et al. 2019). An example of this includes experience with climate policy where governments have originally intended to impose concentrated costs on a relatively small number of politically powerful polluters, resulting in backlash and intense lobbying that has watered down or revoked legislation (Rabe 2018; Jordan and Matt 2014).

In the literature, the policy feedbacks can be used in a deterministic way to reflect the effects of the design and implementation of a specific policy instrument on the likelihood that it will be successfully institutionalised or be undermined and defeated (Jordan and Matt 2014). Individual policy reforms may not immediately lead to a fundamental change in system reconfiguration, and policy feedbacks may also drive more radical policy changes in the future that dramatically reshape social, economic and political conditions (Edmondson et al. 2019). This is because policy feedbacks influence the relationships between actors responsible for policy decisions and various interest groups, including their goals and capabilities, access to resources and institutional arrangements (Edmondson et al. 2019). Over time, reinforcing feedbacks associated with incremental policy reforms could change these relationships so fundamentally that they lead to paradigm shifts and more radical reforms, as documented in the evolution of agricultural policy reforms in the European Union (Daugbjerg 2003). As such, they can bring a system into a ‘critical state’ (or large-scale tipping point) from which it may tip to a qualitatively new state (Stadelmann-Steffen et al. 2021).

An important factor influencing these dynamics is the strength of the coalition supporting the status quo (Patashnik and Zelizer 2009; Edmondson et al. 2019). If policy feedbacks cause a shift in the interests of powerful actors, an existing coalition may become disrupted and weakened and unable to forcefully oppose reform (Daugbjerg 2003). A lack of policy action can also create destabilising feedback effects. For example, social movements demanding policy action (e.g. the recent Fridays for Future movement) have pushed political systems towards criticality through growing bottom-up pressure on policy makers, making it more likely that the system will undergo a major shift (Winkelmann et al. 2022). This suggests ways for actors to weaken the dominant balancing feedbacks that create inertia in existing unsustainable configurations, as well as to generate reinforcing feedbacks that can drive systems towards major reconfigurations (Fig. 1).

## Unlocking transformations to the SDGs—synthesising key insights for acceleration

**Fig. 3:**
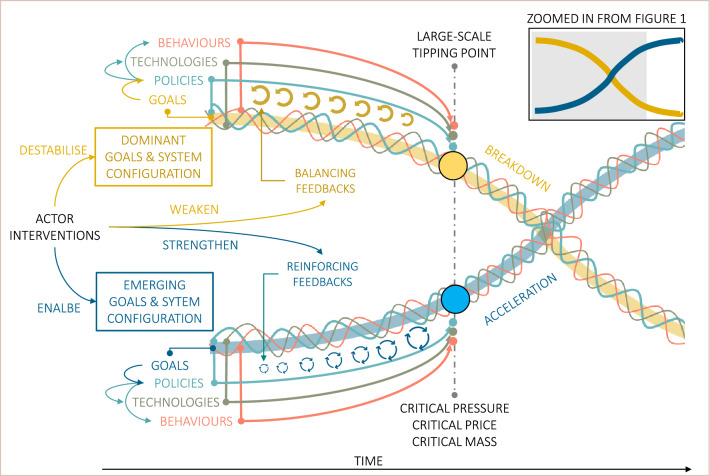
Acceleration of system transformations through shifting system goals, polices, technologies and behaviours. Important system design features for acceleration correspond to new/emerging policies, technologies and behaviours, each of which has several important reinforcing feedbacks and tipping points (blue curve). Important system lock-ins and balancing feedbacks are similarly associated with dominant policy-institutional, techno-economic and social–behavioural feedback mechanisms (yellow curve). Various actors can build enabling conditions to strengthen reinforcing feedbacks and weaken balancing feedbacks (see Table 2). The sequencing of interventions should aim to build momentum over time. The comparative strength of dominant feedbacks can reach a large-scale tipping point, when mutually reinforcing policy, technology and behavioural feedbacks overpower system lock-ins resulting in rapid acceleration. Important tipping points include critical pressure, critical price and critical mass (described in Table 2)

There are many insights from this literature for unlocking and accelerating transformations to the SDGs which can improve the planning and implementation of transformative actions by societal actors. Here, we briefly synthesise important insights stemming from the different literatures, before discussing their implications for actors seeking to accelerate progress towards the SDGs. Building on the earlier Fig. 1 (transition phases and tipping points) and Fig. 2 (nested framework of leverage points), Fig. 3 provides a stylised representation of the emergence and acceleration phases of a system transition moving from a dominant unsustainable system configuration (yellow curve) to an emerging sustainable development system configuration (blue curve). This could represent the transformation of any system that is important for achieving the SDGs, such as health, education, water, sanitation, food, energy, industry, transport or natural systems. The SDGs propose sustainable goals and targets for each of these systems; however, whether or not current system configurations support these sustainable outcomes will vary across contexts.

**Table 2:** Important elements of systems design for acceleration and associated feedbacks, tipping points and enabling conditions

System design	Feedbacks	Tipping points for acceleration	Enabling conditions for feedbacks
Policies and institutions	Socio-political, fiscal and administrative	Critical pressure to take decisive policy action or major reforms	New narratives, problem definitions and framings; mobilising supportive coalitions, constituencies, powerful actors, coordinated lobbies; changing stakeholder access to institutions and policy networks; improved vertical and horizontal coordination; crises, shocks and regime destabilisation; improved administrative capabilities, reputation and morale
Technologies and innovation	Increasing returns to adoption—learning by doing, economies of scale, technological reinforcement; social contagion	Critical price (e.g. price-parity tipping points); critical mass of adopters	Protected spaces for innovation and experimentation; innovation networks and niche lobbies; new business models and access to finance; policies promoting innovation (e.g. R&D, targets, missions, incentives and pilots); maturation of innovations (economic competitiveness, performance, accessibility); policies that give new innovations a competitive edge (e.g. subsidies, tariffs, dedicated markets and supportive infrastructure investment)
Social norms and behaviours	Information cascades; social contagion (positive experience); cognitive feedbacks	Critical mass of adopters or believers/supporters	Positive narratives and experience; access to information; education and awareness; independent media; desirability of alternatives, capacity to adopt; behavioural nudges

The literature highlights that existing system configurations comprise dominant policies and institutions, technologies and infrastructure, and behaviours and social norms which are ultimately oriented towards (or stabilised around) the emergent goals that the system is currently achieving (Fig. 3, yellow curve). We use ‘goals’ in Fig. 3 to represent the system intent, including the beliefs, goals or paradigm of actors that shape the emergent directionality of a system. Nested within this, the existing system design includes the dominant policies, technologies, behaviours and corresponding feedbacks which stabilise around the dominant goals (also depicted in Fig. 3). Both dominant ‘unsustainable’ system configurations (Fig. 3, yellow curve) and emerging ‘sustainable’ system configurations (Fig. 3, blue curve) could thus be thought to comprise their own sets of goals, policies, technologies and behaviours. Balancing feedbacks (Fig. 3, ) are dominant in stabilised systems and generate inertia and resistance to change, while reinforcing feedbacks (Fig. 3, ) can unlock rapid shifts between system states when they reach critical points.

To improve system performance, common interventions from policy makers and other actors include adjusting parameters associated with system design elements (e.g. through subsidies and incentives). Such interventions are thought to have limited transformative effect as they target a low point of leverage (Fig. 2**,** ‘parameters’); however, they could build momentum over time if they are oriented towards new transformative goals. Interventions may also include more decisive reforms that fundamentally change system design features and reorient systems towards new goals (e.g. through legislated targets, regulatory bans and large-scale public investments).

Interventions can better contribute to systems change if they weaken balancing feedbacks and strengthen reinforcing feedbacks (Fig. 3). Rapid shifts in systems commonly occur once tipping points are crossed which results in the acceleration of the new and the breakdown of the old, for example, when a new technology reaches a critical mass of adopters and rapidly diffuses and replaces an existing technology. However, diffusion of single innovations may not be sufficient to tip a whole system towards fundamentally new goals. To trigger such a fundamental shift in whole system configuration, it may be necessary to cross multiple tipping points associated with policy, technology and behavioural feedbacks—what we note as a ‘large-scale tipping point’ (Fig. 3)—which could then compel a system to reorient around new goals.

In the context of the SDGs, shifting the overall goals and intent of systems to reorient them towards the SDGs represents an obvious strategic intervention with deep leverage potential. However, the SDGs are voluntary and changes in stated goals will have little effect if systems design and feedbacks are not adapted to align to these new goals (Kieft et al. 2020). Recent evidence suggests that while countries have adopted and localised the SDGs to varying degrees in national strategies and reports, they have had limited impact thus far in fundamentally changing systems design such as through new policies and institutions (Biermann et al. 2022; Allen et al. 2021). This suggests that unsustainable goals of existing systems (whether implicit or explicit) continue to dominate in many countries and systems.

Fundamental shifts in emergent system goals will face considerable resistance, particularly where there are highly divergent views, powerful vested interests or a lack of trust or social cohesion. As such, actors seeking to embed the SDGs in a way that reorients systems will need to strategise, build momentum and look for opportunities at shallower points of leverage to shift systems towards these goals. The emerging literature on tipping points and leverage points suggests that these opportunities exist in rewiring systems design so that important system feedbacks create the conditions for acceleration, compelling systems to change. However, it is also important that transformative goals and intent remain in clear focus, so that changes build towards these. In this context, we suggest three broad strategies for would-be reformers seeking to unlock transformations to the SDGs.

First, actors should determine important systems to be transformed (e.g. health, energy and food), envision and localise new system goals aligned to the SDGs, and begin to build momentum towards these new goals. This would include clarifying the existing (implicit or explicit) goals that drive current system performance as well as desirable new system goals aligned with the SDGs and suitable to local contexts. Where these current and new goals are similar, then smaller adjustments to systems design may be sufficient. However, where these goals are fundamentally different, actors should seek to clarify important system design elements needed to support these new goals, including new policies, technologies and behaviours. In this initial phase, they should also start to build a convincing narrative and broad coalition around new goals and design features and encourage support from powerful actors.

Second, actors should seek to identify important system feedbacks and augment systems design in ways that shift feedbacks in favour of new goals. This can be done by building knowledge of common policy, technology and behavioural feedbacks that govern inertia and non-linear dynamics in systems and their tipping points and enabling conditions (Table 2). Systems inertia is commonly associated with techno-economic, political–institutional and social-behavioural lock-ins (Klitkou et al. 2015; Geels 2019) which have a stabilising effect on systems and often become optimised and strengthened over time. Similarly, reinforcing feedbacks are associated with new policies and institutions, new technologies and innovations, and new behaviours and social norms (Stadelmann-Steffen et al. 2021; Farmer et al. 2019). Actors can seek to identify opportunities to reform existing system design in ways that weaken balancing feedbacks that stabilise unsustainable configurations (Fig. 3, ) and strengthen reinforcing feedbacks that favour emerging sustainable configurations (Fig. 3, ). Implementation efforts can start with easier or more feasible interventions that augment these feedbacks without triggering strong resistance (Lenton et al. 2021; Kieft et al. 2020). Actors should also identify more decisive policies and actions that could trigger acceleration but that face strong resistance or major impediments.

Third, actors should use well-sequenced and timed interventions to build enabling conditions and momentum for decisive government action that can trigger large-scale tipping points and accelerate the shift to sustainable systems configurations oriented around the goals. Governments will not act decisively unless the right enabling conditions are in place, which include supportive coalitions, pervasive narratives, mass public support and maturing technologies (Markard et al. 2016; Hess 2014; Meijerink 2005; Meckling et al. 2015; Raven et al. 2016; Roberts and Geels 2019). Actors can seek to build these enabling conditions in systems to unlock powerful feedback effects. The timing (Patashnik and Zelizer 2009) and sequencing (Meckling et al. 2017) of interventions are critical to build momentum for major systems change over time (Edmondson et al. 2019). Actors can gradually build momentum and await opportune moments to push for more decisive policies and reforms, such as crisis events, shifts in powerful actors, changes in government or escalating bottom-up pressure.

### Building enabling conditions for important system feedbacks to unlock acceleration

An important finding from the literature is that while a key objective of those seeking transformations to the SDGs is to reconfigure systems around new desirable goals, such deep points of leverage face strong resistance. Faced with such resistance and inertia, actors can build enabling conditions for important system feedbacks that can push systems towards ‘large-scale tipping points’ (Fig. 3). In Table 2, we briefly summarise findings from the literature relating to important enabling conditions associated with common policy, technology and behavioural feedbacks and which can be targeted by actors. Here we further discuss the nature of these feedbacks and how policymakers and other actors can build enabling conditions to trigger large-scale tipping points and acceleration towards the SDGs.

Enabling conditions for *policy feedbacks* (Table 2) include the mobilisation of coalitions, powerful actors and coordinated lobbies in support of change (or the status quo) which can exert pressure on policymakers to take decisive action (or take no action) (Markard et al. 2016; Hess 2014; Meijerink 2005; Meckling et al. 2015; Raven et al. 2016; Roberts and Geels 2019). Policy feedbacks generate political support and coalitions by providing resources and incentives that result in powerful constituencies willing to strenuously protect those resources. This can create system lock-ins (Fig. 3, yellow curve) where existing policies, regulations, standards and networks favour existing coalitions and incumbents and create an uneven playing field for new entrants. Dominant coalitions can also use their superior resources, lobbying influence and privileged access to policy networks to overturn or water down policy reforms. Policy feedbacks can also be reinforcing (Fig. 3, blue curve) if they support new constituencies and coalitions in favour of change, alter perceptions and behaviours, expand new state capacities and budgetary allocations, and reconfigure institutional structures and policy networks to allow new actors and shift power balances (Jacobs and Weaver 2015; Kern et al. 2019; Edmondson et al. 2019).

Actors can nurture beneficial policy feedback effects through interventions that erode the financial resource base, legitimacy and political support of dominant coalitions and incumbent actors (Rosenbloom 2018; Roberts 2017). This could include divestment, removing favourable subsidies or limiting access to policy networks. Resistance can also be weakened through complementary policies targeting those who are negatively impacted by transitions (Fesenfeld et al. 2020), such as providing compensation (e.g. redundancy payments and early retirement benefits), social safety nets or assisting reorientation (e.g. skills upgrading, retraining, alternative employment and regional innovation or development policies) (Spencer et al. 2018). Incentives can also be provided to incumbents who are willing to innovate and adapt to the new sustainable configurations (Kivimaa et al. 2019; Rogge and Johnstone 2017). Policy actors can also actively nurture reinforcing policy feedbacks by introducing policies that build new coalitions, provide access to policy networks and stimulate public debate, which can gradually build political momentum and create the conditions for stronger or more radical policies (Geels 2019). In terms of accelerating transitions, policy feedbacks can reach a ‘critical pressure’ tipping point when ‘coalitions for change’ overcome ‘coalitions for resistance’, providing the political feasibility needed for governments to take decisive policy action in favour of sustainable development (Table 2, Fig. 3).

Enabling conditions for *technology feedbacks* (Table 2) include the maturation and increased competitiveness, performance and accessibility of new innovations which provides feasible solutions that policymakers can push (Markard et al. 2016; Schmidt and Sewerin 2017; Sharpe and Lenton 2021; Otto et al. 2020a). Technology feedbacks include increasing returns to adoption, learning by doing, economies of scale and technological reinforcement which can both hinder and promote acceleration (Sharpe and Lenton 2021; Lenton et al. 2021). Important technology tipping points include critical price (e.g. of a new sustainable versus incumbent technology) and critical mass of adopters of a new technology (Sharpe and Lenton 2021; Lenton et al. 2021; Zeppini et al. 2014) (Table 2). Technology feedbacks can create system lock-ins (Fig. 3, yellow curve) where the low cost and high-performance characteristics of existing technologies (e.g. coal-fired generators and internal combustion vehicles) give them a strong competitive advantage over emerging sustainable alternatives. However, these feedbacks can also reinforce (Fig. 3, blue curve) new emerging technologies (e.g. solar PV and electric vehicles), particularly once they cross important tipping points in terms of economic competitiveness, performance and accessibility.

Policymakers and other actors can build enabling conditions to harness technology feedbacks through investment in R&D, pilot projects, missions, public procurement, subsidies, feed-in tariffs, dedicated markets and infrastructure investment (Meckling et al. 2017; Roberts and Geels 2019; Markard et al. 2020; Geels 2019). Policies that improve the competitiveness of alternative technologies can help to bring forward price-parity tipping points and trigger acceleration (Sharpe and Lenton 2021; Broadbent et al. 2022). However, given the diverse country contexts and the geographic bias of empirical studies towards the Global North, countries may first need to consider and resolve impediments to the emergence and diffusion of innovations, including weak institutions and innovation systems, and market imperfections (Ramos-Mejía et al. 2018; Hansen et al. 2018; Wieczorek 2018).

Enabling conditions for *behavioural feedbacks* (Table 2) include pervasive narratives and increased awareness which may shift social norms and behaviours, generate mass public support, legitimise stronger policy action, or discredit incumbent actors and provision of policy support (Roberts and Geels 2018; Rosenbloom et al. 2016; Kern et al. 2014). Behavioural feedbacks include social contagion effects and cognitive feedbacks, and tipping points can be reached through a critical mass of adopters or supporters (Table 2**, **Fig. 3). Behavioural feedbacks can create inertia in existing systems (Fig. 3, yellow curve) when social norms and lifestyles become organised around particular practices and behaviours which can create lock-ins, whereby existing practices are more desirable or socially acceptable and normalised across stakeholders (Klitkou et al. 2015). While preferable alternative behaviours and norms may exist, knowledge and awareness of these, their desirability and general convenience may be lacking. However, social contagion feedbacks can also have reinforcing effects (Fig. 3, blue curve), where new behaviours, practices and norms spread through information networks as a result of positive experiences and imitation of others. A critical mass tipping point may be crossed (e.g. around 20–30%) resulting in rapid acceleration and widespread adoption of new practices, ideas and behaviours (Centola et al. 2018; Rogers 1962; Lenton et al. 2021).

Policymakers and other actors can promote the adoption of new social norms and behaviours by increasing access to information (e.g. marketing and communication campaigns) and independent media, formal education, enhancing information networks, providing financial incentives or rewards, and through behavioural nudges (Rosenow et al. 2017; Creutzig et al. 2018). Policymakers may also phase out undesirable behaviours over time, for example through media campaigns or more stringent regulation or bans, as seen in the case of tobacco, gambling or firearms.

What emerges from this discussion of different types of system feedbacks is that they are mutually reinforcing and together build important enabling conditions that can trigger a large-scale system tipping point (Fig. 3). Policymakers and other actors can play a cross-cutting role, supporting new technologies, behaviours and social norms and building coalitions and political momentum for major reforms, while also destabilising and disrupting the current configuration. Early targeted interventions such as investing in R&D and subsidies and incentives for sustainable technologies and practices may face less political resistance and could fast-track technological improvements and create economic conditions to rapidly scale alternatives. This could pave the way for more stringent policies that have played a decisive role in accelerating past transitions, such as taxes or pricing mechanisms, large-scale public infrastructure investments, guaranteed markets with fixed prices and substantial investment grants (Roberts and Geels 2019). Such reforms may be more feasible when shocks or crises weaken resistance of incumbent organisations or create pressure for fundamental change (Roberts and Geels 2019; Capoccia and Kelemen 2007; Avelino et al. 2016; Gunderson et al. 2017; Herrfahrdt-Pähle et al. 2020). High leverage interventions could simultaneously weaken balancing feedbacks while strengthening reinforcing feedbacks, such as shifting existing subsidies from incumbents to alternatives (‘subsidy swaps’), divesting from incumbents and reinvesting in sustainable technologies and businesses (‘investment swaps’), and by altering policy networks to exclude incumbents and include new actors (‘access swaps’) (Barbier and Burgess 2020; Otto et al. 2020a).

Placing this in the context of the SDGs, while all the feedbacks are likely important for any system transition, their comparative influence or importance will vary across different systems. Technology feedbacks may be more important in systems transitions that are heavily dependent on new technologies, such as in energy, urban or transport systems (Otto et al. 2020a; Farmer et al. 2019; Sharpe and Lenton 2021). Policy feedbacks are likely important in all systems, but particularly prominent in systems transitions involving major social policy change, for example in social protection systems or universal healthcare. Behavioural feedbacks may be particularly important in lifestyle changes associated with health, food or waste systems, such as phasing out unhealthy or highly consumptive practices (e.g. tobacco, alcohol, gambling, meat and fast fashion) or adopting healthy practices (e.g. regular exercise, healthy diets and recycling) (Richardson 2012).

## Conclusions, limitations and areas for further research

A broad range of systems transformations will be needed to achieve the SDGs, including for the provision of healthcare, education, food, energy, mobility, housing, water, among others. In the literature, such transformation involves a fundamental change in system goals and paradigm, while transition is the process of systems change over time. Successful transformations proceed in three phases taking the shape of an S-curve, with a slow emergence phase followed by the rapid acceleration of new ideas, practices and innovations which then stabilise in a reconfigured system state aligned to new goals. The slow progress on the SDGs to date has brought increased focus on how to trigger the acceleration phase and move more quickly towards the goals.

Different countries are at varying stages of transition and have different starting points and priorities. As such, there is no single blueprint for accelerating transformations. However, there is an expanding knowledge base on important dynamics, impediments and enabling conditions across diverse literatures. This knowledge can help to inform actors seeking to influence the speed and direction of transitions towards the SDGs, by identifying strategic interventions that leverage important system feedbacks that result in rapid, large-scale systems change. The emerging literature on tipping points and leverage points suggests that opportunities exist in rewiring systems design so that important system feedbacks create the conditions for acceleration, while ensuring that transformative goals and intent remain in clear focus.

Shifting the goals and intent of systems to reorient them towards the SDGs represents an obvious strategic intervention with deep leverage potential but faces strong resistance due to diverse policy, technology and behavioural lock-ins. Actors will need to build momentum to reorient systems around new goals. This can be done by building knowledge of common policy, technology and behavioural feedbacks that govern systems inertia and acceleration dynamics along with their tipping points and enabling conditions. Where resistance is strong, actors can seek to augment systems design in ways that weaken balancing feedbacks associated with existing system configurations and strengthen reinforcing feedbacks associated with emerging new system configurations oriented around the SDGs. Governments have a critical role to play in triggering acceleration through decisive policy action; however, a range of actors can put in place critical enabling conditions to augment system feedbacks and build momentum over time, including by supporting innovation and behaviour change and building and maintaining political support for change throughout the transition process. This can push systems towards large-scale tipping points and trigger acceleration to new systems configurations.

However, several characteristics of the SDGs make them particularly demanding for research on transformations including their comprehensive scope, future orientation, indivisibility, universality and voluntary nature. The very broad and comprehensive scope of sectors, systems and targets addressed by the SDGs highlights two important gaps in current research. First, transitions in energy, food and urban systems tend to dominate the literature with a strong focus on technology innovations, while other systems related to social policy such as social protection, education and health systems receive less attention. This generates a potential systems bias in the literature and limits the transferability of insights across sectors with different characteristics.

Second, much of the research on transitions has focussed on single systems (or even single innovations within systems) and there are few insights into multi-system interactions. The potential trade-offs and synergies between the SDGs are a well-known characteristic; however, there has been little research on how these play out dynamically during simultaneous transitions across multiple systems, or how they should be effectively managed.

A third gap in the literature relates to country context. While the SDGs are universal and apply to all countries, much of the research to date has focussed on transformations in the developed world. Emerging studies on transitions in developing countries highlight that they face a range of additional capacity constraints which are likely to significantly hamper acceleration, including important administrative and institutional capacities, science and innovation capabilities, and undeveloped markets and financial sectors. These factors will likely present additional lock-ins for transformation in developing countries which need to be further explored to identify potential solutions.

Finally, while the literature acknowledges that transformations involve fundamental shifts in goals and paradigms, there remains limited practical guidance on how such shifts can be deliberately brought about or accelerated. Emerging research on ‘deep transitions’ suggests that major paradigm shifts do occur and may even be inevitable. However, they may not be realistic in the 2030 timeframe associated with the SDGs. Further research is needed on how paradigm shifts can be deliberately accelerated by actors, and how important shifts within systems can propagate across systems to change the directionality of all systems towards sustainable development.

## Data Availability

There are no data associated with the manuscript.

## Footnotes

1 TS = (sdg* OR “sustainable development goal*” OR “sustainable development”) AND TS = ("transformation*" OR "transition*") AND TS = (“lever*” OR “leverage point*” OR “tipping point” OR accelerat* OR feedback OR “policy mix*” OR “policy process*” OR “system dynamic*”) > 529.
